# Cell cycle visualization tools to study cardiomyocyte proliferation in real-time

**DOI:** 10.1098/rsob.240167

**Published:** 2024-10-09

**Authors:** Rustem Salmenov, Christine Mummery, Menno ter Huurne

**Affiliations:** ^1^ Department of Anatomy and Embryology, Leiden University Medical Center, Leiden 2300RC, The Netherlands; ^2^ The Novo Nordisk Foundation Center for Stem Cell Medicine (reNEW), Leiden University Medical Center, Leiden 2300RC, The Netherlands

**Keywords:** cardiomyocyte proliferation, live cell cycle reporter, human iPSC-derived cardiomyocytes, 3D cardiac organoids

## Abstract

Cardiomyocytes in the adult human heart are quiescent and those lost following heart injury are not replaced by proliferating survivors. Considerable effort has been made to understand the mechanisms underlying cardiomyocyte cell cycle exit and re-entry, with view to discovering therapeutics that could stimulate cardiomyocyte proliferation and heart regeneration. The advent of large compound libraries and robotic liquid handling platforms has enabled the screening of thousands of conditions in a single experiment but success of these screens depends on the appropriateness and quality of the model used. Quantification of (human) cardiomyocyte proliferation in high throughput has remained problematic because conventional antibody-based staining is costly, technically challenging and does not discriminate between cardiomyocyte division and failure in karyokinesis or cytokinesis. Live cell imaging has provided alternatives that facilitate high-throughput screening but these have other limitations. Here, we (i) review the cell cycle features of cardiomyocytes, (ii) discuss various cell cycle fluorescent reporter systems, and (iii) speculate on what could improve their predictive value in the context of cardiomyocyte proliferation. Finally, we consider how these new methods can be used in combination with state-of-the-art three-dimensional human cardiac organoid platforms to identify pro-proliferative signalling pathways that could stimulate regeneration of the human heart.

## 1. Cardiomyocyte turnover in disease and development

Cardiovascular diseases are the leading cause of mortality and morbidity worldwide, accounting for approximately 19 million deaths in 2020, an increase of almost 20% from 2010 [1]. One of the most prevalent heart pathologies is ischaemic heart damage, caused by coronary artery blockage. The lack of oxygen and nutrients results in extensive cardiomyocyte (CM) death, formation of scar tissue and reduced contractility of the heart [2,3]. Adult CMs have inherently low turnover rates in ageing hearts, ranging from <1% to 4% per year [4–9] and this does not significantly increase in pathological conditions [10–12]. As a result, and by contrast to organs like skin, endogenous CMs cannot replace those lost after damage, and functional recovery does not take place. By contrast, CMs proliferate vigorously during heart development [13] and this continues in rats and mice, albeit at a lower rate, for a short period after birth [4,14]. This is also evidenced by the high regeneration potential of the mouse heart from postnatal day (P)1 until P7 after resection of the left ventricle or myocardial infarction (MI) [15,16]. Moreover, cardiac cell proliferation and the survival and recovery of neonatal mice occur even after ablation of 50% to 60% of their hearts [17]. In line with these findings, functional recovery post-MI has also been observed in the neonatal human heart [18–20], although the exact contribution of CM proliferation has not been determined.

## 2. Cell cycle features of cardiomyocytes

The decline in CM cell cycle activity after the first postnatal week [8,14] coincides with their transcriptional, structural, functional and metabolic maturation [13,21]. Throughout maturation, CMs express decreasing levels of pro-proliferative transcription factors, cyclins, and cyclin-dependent kinases (CDKs) [14,21–23]. On the other hand, the expression of CDK-inhibitors and pocket proteins, which prevent cell cycle progression [24,25], increases and presumably facilitates CM cell cycle arrest [26,27]. The specific cell cycle stage at which CMs finally arrest is, however, not entirely clear but seems to differ between humans and mice. Mice studies suggest that the majority of adult CMs are arrested in the G1-phase or early S-phase both *in vivo* as well as *in vitro* [28,29]. Human CMs derived from induced pluripotent stem cells (iPSC-CMs), on the other hand, were shown to arrest in the late S- [30] or G2-phase [31]. As distinct molecular checkpoints are responsible for cell cycle arrest at the G1-, S- and G2/M-phase [32,33], these interspecies differences might have important implications for therapeutic approaches that aim to promote CM proliferation by overcoming these checkpoints. Moreover, the different experimental approaches and markers used to determine cell cycle phase cause ambiguity with respect to the phase in which non-proliferating CMs arrest.

This is further complicated because CMs in the maturing heart can undergo alternative types of cell cycle events, such as endoreplication and endomitosis [34]. Endoreplication is caused by the failure in karyokinesis (nuclear division) and results in a single nucleus containing an increased number of chromosomal pairs, also known as polyploidy [34]. Endomitosis, on the other hand, is the formation of multiple nuclei through incomplete cytokinetic division. As a result, the proportion of binucleated CMs increases with age [34], reaching up to 95% of the total CM population in adult mice [35]. Multinucleation contributes to the very large increase in CM size and volume, which facilitates the transition from hyperplastic to hypertrophic growth in the maturing heart. This is needed to meet the physiological demands of a growing organism [36]. Postnatal CM multinucleation and polyploidization occur over different time frames across different species, taking approximately 3 weeks after birth in mice and decades in humans [34]. Although multinucleation and polyploidization play pivotal roles in controlling organ growth and function, they also contribute to the cessation of CM proliferation [37,38] and, hence, impede heart regeneration in zebrafish and mice as they age [39,40]. The negative effect of multinucleation on proliferation was reflected in the comparative transcriptional analysis of mononucleated and binucleated CMs at P7; this revealed an inverse relationship between binucleation and expression of proliferation genes [41]. Furthermore, this report also indicated transcriptional differences between mononucleated and binucleated adult CMs, although a similar study found no clear transcriptional differences between mono- and multinucleated CMs from the adult mouse heart [42]. The prevalence of CMs with different degrees of nucleation and ploidy varies between species. In adult mouse hearts, the largest population of all CMs are binucleated diploid or binucleated polyploid [34], and even tetraploid mononucleated and tetraploid binucleated CMs were observed [35]. By contrast, roughly 70% of CMs in adult human hearts are mononucleated, and with ageing, the average DNA content per nucleus increases by more than 1.5-fold due to polyploidization [8,38]. These interspecies differences in the CM ploidy probably affect how CMs respond to pro-proliferative signals and to what extent these signals can promote heart regeneration, emphasizing the importance of human models for their higher predictive value and therefore importance in identifying pro-regenerative stimuli for the human heart.

## 3. Limitations of antibody-markers to study CM proliferation

As the induction of CM proliferation is among the therapeutic approaches being considered to treat heart failure, knowledge of the molecular pathways that control CM cell cycle progression and/or arrest is crucial for the development of regenerative therapies. Despite substantial efforts, there are few studies visualizing CM cell cycle progression and little direct evidence of cell cycle division. Traditionally, studies have relied on the antibody-based detection of proteins that are expressed during specific cell cycle phases (table 1). The most commonly used cell cycle markers include, for example, the synthetic nucleoside analogues 5-bromo-2′- and 5-ethynyl-2′- deoxyuridine (BrdU and EdU, respectively). These structural thymidine analogues are incorporated during DNA replication and thus label cells actively progressing through the S-phase [43,44]. Although highly specific for S-phase cells, prolonged exposure of cells to thymidine analogues has been associated with genotoxicity and disruption of cell division, hampering their use in longitudinal studies [44–46]. Moreover, BrdU incorporation coincides with DNA damage repair, without the inducing replication [47]. Together, these issues could lead to incorrect claims of proliferation, in particular in conditions where DNA damage is induced. The predictive value of this method is therefore limited, especially when used in high-throughput screening setup to test large numbers of conditions/compounds.

**Table 1.** Cell cycle markers and their characteristics for studying cardiomyocyte proliferation.

Cell cycle marker	Phase	Characteristics
PCNA	G1 and S	Marks proliferating cells
		Does not depict mitosis
		Is not suited for live cell imaging
EdU/BrdU	S	Marks S-phase progression
		Long-term exposure disrupts cell cycle progression
		Does not depict mitosis
		Is not suited for live cell imaging
Ki-67	S and G2/M	Marks proliferating cells
		Does not indicate transitions between phases
		Does not discriminate mitosis from alternative cell cycle events
		Is not suited for live cell imaging
AurkB	G2/M	Marks late mitotic events
		Does not discriminate mitosis from failed cytokinesis
		Is not suited for live cell imaging
		Is challenging to detect
pHH3	G2/M	Marks late mitotic events
		Does not distinguish mitosis from alternative cell cycle events
		Is not suitable for live cell imaging

AurkB, aurora kinase B; BrdU, 5-bromo-2'-deoxyUridine; EdU, 5-ethynyl-2'-deoxyuridine; PCNA, proliferating cell nuclear antigen; pHH3, phospho-Histone H3.

Ki-67 expression is also frequently used to distinguish proliferating from quiescent cells [48,49]. Although Ki-67 is generally believed to be absent in quiescent cells, a recent study indicated that cells in the early stages of quiescence still express Ki-67, which could potentially bias the results of studies aimed at the identification of factors that have (lasting) pro-proliferative effects [50]. Like Ki-67, the DNA replication processivity factor proliferating cell nuclear antigen (PCNA) is mainly expressed during the S-phase and is therefore widely used as a marker for proliferating cells [51,52]. It is important to note, however, that both Ki-67 and PCNA play important roles in DNA damage repair and that their expression levels are affected by conditions that induce DNA damage [53]. As with the incorporation of thymidine analogues, this could also lead to false positive results and should be considered while interpreting the results of high-throughput screening experiments.

Besides these limitations, antibody staining of these markers can only identify cells in the S-phase and are unable to visualize the transition to the G2/M-phase. For this, markers of the G2/M-phases have been identified, such as the aurora kinase B (AurkB) and phospho-Histone H3 (pHH3). AurkB facilitates cytokinesis by ensuring the integrity of microtubule attachment to the kinetochore during midbody formation [54] and is therefore expressed specifically in the G2/M-phase. Similarly, phosphorylation of Histone H3 is required for chromatin condensation and is consequently crucial for cell cycle progression through the late G2/M-phase [55]. Although these markers are conventionally used as a proxy for cell proliferation, assuming that cells that overcome the restriction point and enter the S-phase will complete the cell cycle and divide, they do not discriminate between CM division and alternative cell cycle events such as endoreplication or endomitosis [56]. The inability to distinguish these processes can lead to potentially controversial claims about CM proliferation. It has been shown, for example, that Ki-67, can be expressed in CMs arrested in the G2-phase [56]. Along these lines, the detection of pHH3-positive nuclei and AurkB occurs at the M-phase but is unable to make distinctions between cell division and failed cytokinesis [56–58]. Therefore, while these markers indicate the cell cycle activity of CMs, they cannot serve as direct proof of CM division.

## 4. Live fluorescent cell cycle reporters

### 4.1. Fluorescent intensity-based systems

**Figure 1.**
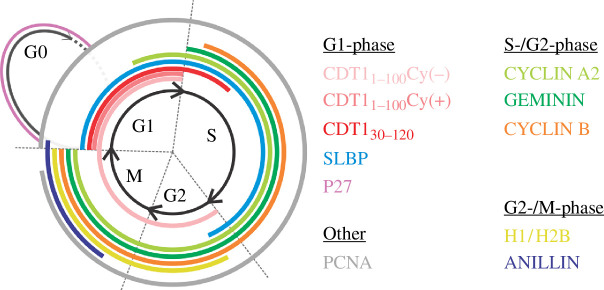
Fluorescent-intensity-based systems. Expression of cell cycle reporters in the different phases of the cell cycle. Abbreviations: Cy, cyclin-CDK recognition motif; H1/H2B, Histone 1 and Histone 2B; PCNA, proliferating cell nuclear antigen; SLBP, stem loop histone mRNA binding protein.

As an alternative to antibody-based staining, live cell cycle reporter systems were developed to facilitate robust identification of dynamic cell cycle changes. The fluorescent ubiquitination-based cell cycle indicator (FUCCI) system was one of the first developed to track cell cycle progression in live cells [59]. FUCCI is based on the controlled expression of a pair of fluorescent probes, monomeric Kusabira Orange 2 (mKO2) and monomeric Azami Green (mAG), fused to truncated versions of Cdt1 and Geminin, respectively. These proteins play important roles during different phases of the cell cycle [60–62] and their expression is controlled by the reciprocal activity of two E3 ubiquitin ligases, SCF^SKP2^ and APC^Cdh1^, respectively. SCF^SKP2^ is predominantly expressed during the S- and G2-phase and mediates the targeted degradation of Cdt1 through the recognition and ubiquitination of its Cy motif [63,64]. APC^Cdh1^, on the other hand, induces the degradation of Geminin at the late M-phase [65,66]. By monitoring the cyclic expression of Cdt1_30-120_–mKO2 and Geminin_1-110_–mAG transitions between the G1- and S-/G2-phases can be observed. Moreover, the G1- to S-phase transition can be visually captured as an overlap of both fluorescent signals [59] (figure 1).

Since its development, the FUCCI reporter system has provided a major contribution to understanding cell cycle-regulated processes. The original FUCCI reporters have been used in a variety of cell lines to study proliferation, progression through cell cycle checkpoints, distinguishing proliferating and quiescent cells, and monitoring variability in cell cycle duration [67,68]. The FUCCI system has revealed the signalling pathways that shape the cell cycle of mouse embryonic stem cells (ESCs) [69,70], how the cell cycle state affects the differentiation potential [71] and how certain proteins control not only cycle progression but also cell fate decisions [72]. The FUCCI system was also used to study CM proliferation in transgenic zebrafish, *Drosophila* and mice [28,73–75]. Nevertheless, despite being widely used for studying the cell cycle, the original FUCCI system is not able to clearly distinguish between G0- and G1-phase as the Cdt1 reporter is expressed in both phases [59]. Moreover, Geminin expression increases from the S-phase and declines in the late G2-/M- phases, hence the system cannot discriminate between S- and G2-/M-phases either [59]. Several adjustments have been made to the FUCCI system to circumvent these limitations and improve the separation of different cell cycle phases (table 2).

**Table 2.** Summary of cell cycle visualization systems to study cardiomyocytes proliferation in real time.

	Reporter system	Advantages	Limitations
**fluorescent-intensity based systems**	FUCCI	Clear discrimination between G1- and S-/G2-phase	Unable to distinguish S- from G2-/M-phase
		Widely used across different cell types and animal models	Does not visualize karyokinesis
		Cardiac-specific constructs are available	
	FUCCI(P27(CDK^−^))	Identifies cells transitioning from G0- to G1- phase	Unable to distinguish between S- and G2-/M-phase
		Useful for studying quiescence in CMs	Does not visualize karyokinesis
	FUCCI(CA)	Enhanced accuracy in visualizing G1- to S- and S- to G2-phase transitions	Fluorescent intensity is affected by DNA damage
			Does not visualize karyokinesis
	FUCCI(SCA)	Clear discrimination between G1- and S-/G2-phase cells	Unable to distinguish S- and G2-/M-phase
			Does not visualize karyokinesis
			Fluorescent intensity is affected by DNA damage
	PIP-FUCCI	Visualizes G1- to S- and S- to G2-phase transitions	Does not visualize karyokinesis
	FUCCI4	Discriminates all four cell cycle phases	More complex to implement than the original FUCCI system
		Visualizes histone separation during karyokinesis	
	FUCCI-Red	Uses a single fluorescent channel for cell cycle analysis	Requires advanced imaging setups for lifetime-based analysis
		Advantageous for deep tissue imaging	Does not visualize karyokinesis
	LT-FUCCI	Uses a single fluorescent channel for cell cycle analysis	Requires advanced imaging setups for lifetime-based analysis
		Multiplexing possibilities	Does not visualize karyokinesis
	FUCCI4 with psFPs	Multiplexing possibilities	Dependent on stability and fluorescent properties of psFPs
		Discriminates all four cell cycle phases	Temporal multiplexing can be slow
**localization-based systems**	Cyclin B and A2	Localization depends on cell cycle phase	Functional diversity can complicate the interpretation
		Can be combined with other fluorescent systems	Does not visualize karyokinesis
	CDKs	Can be combined with other fluorescent systems	Functional redundancy of CDKs complicates interpretation
			Does not visualize karyokinesis
	PCNA	Discriminates all four cell cycle phases	Requires advanced imaging setup to accurately distinguish the transitions between phases
		Multiplexing possibilities	
		Can be combined with other fluorescent systems	
	H1/H2B	Visualizes cell cycle events in M-phase	Requires additional reporters for complete cell cycle tracking
		Can be combined with other fluorescent systems	
	Ki−67	Visualizes the M-phase	Requires additional reporters for complete cell cycle tracking
		Useful for studying quiescence and karyokinesis in CMs	Expression in G0- and G1-phases is heterogeneous between cells
	Anillin	Distinguishes cytokinesis from binucleation	May be challenging to detect
		Can be used in combination with physical cues such as midbodies symmetry and nuclei distance to identify cellular division	
**genetic tracing systems**	MADM	Identifies mitotically active cells	Low labelling efficiency
		Useful for animal studies on CM renewal	Does not directly monitor changes in specific cell cycle phases
	AurkB	Identifies mitotically active cells	May overestimate cytokinesis
		Valuable for studying CM proliferation dynamics during heart development and regeneration	Does not directly monitor changes in specific cell cycle phases

AurkB, aurora kinase B; CA, CUL4^Ddb1^-APC^Cdh1^; CDK, cyclin-dependent kinase; FUCCI, fluorescent ubiquitination-based cell cycle indicator; H1/H2B, Histone 1 and Histone 2B; LT, LifeTime; MADM, mosaic analysis with double markers; PCNA, proliferating cell nuclear antigen; PIP, PCNA-interacting protein; psFP, photoswitchable fluorescent protein; SCA, SCF^SKP2^-CUL4^Ddb1^-APC^Cdh1^ .

Co-expression of fluorescently labelled mutant P27 with inactive CDK binding domain (P27(CDK^−^)) for example, contributed, to better discrimination between cycling and non-cycling cells [76]. Although both Cdt1 and P27(CDK^−^) are expressed during the G1-phase, the difference in their degradation kinetics allows the identification of cells transitioning from the G0- to the G1-phase [76]. Because of these unique properties, the P27(CDK^−^) reporter could be a valuable tool in studies aimed at understanding how, and to what extent, cell cycle quiescence contributes to the limited proliferation of CMs.

The FUCCI system was further improved to determine transitions between cell cycle phases. The system was fine-tuned to create FUCCI(SCA) and FUCCI(CA), in which the SCF^SKP2^, CUL4^Ddb1^ and APC^Cdh1^ E3 ubiquitin ligases are responsible for the degradation of fluorescent reporters [77]. In the original FUCCI system, Cdt1_30-120_ degradation kinetics were delayed at the end of the G1- phase and the beginning of the S-phase, resulting in a short period of double fluorescence (early S-phase). Both FUCCI(CA) and FUCCI(SCA) overcome this limitation and can accurately visualize the G1- to S-phase transition [77]. Compared with the original FUCCI reporter, in which a Cy motif within Cdt1_30-120_ is recognized by SCF^SKP2^, the FUCCI(SCA) system uses an alternatively truncated version of Cdt1, namely Cdt1_1-100_, which contains the Cy motif as well as a PIP motif (figure 1). During S-phase, CUL4^Ddb1^ recognizes the PIP motif and keeps Cdt1_1-100_ degraded, while SCF^SKP2^ maintains its degradation during the G2-/M-phases. The presence of both PIP and Cy motifs allows CUL4^Ddb1^ and SCF^SKP2^ to clearly distinguish the G1-phase from the S-, G2- and M-phases. The FUCCI(CA) system uses Cdt1_1-100_ with only the PIP motif, allowing Cdt1 expression to be restored in the G2-/M-phases (figure 1). This system provides a clear distinction between the transitions from the G1- to S-phase and from S- to G2-/M-phases [77]. Similar to the FUCCI(CA) and FUCCI(SCA) systems, Grant *et al*. developed the PIP-FUCCI to precisely pinpoint G1/S and S/G2 transitions by using the CUL4^Ddb1^ targeted degradation of human Cdt1_1-17_ [78]. These strategic modifications resulted in an optimized live cell cycle marker with improved accuracy in cell cycle phase identification.

**Figure 2.**
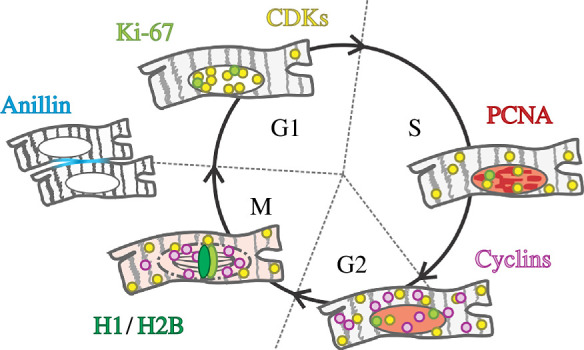
Localization-based markers facilitate the identification of cell cycle phases. The G1-phase is marked by CDKs accumulation in the nucleus, which translocate into the cytoplasm during S- and G2-phases. The S-phase is characterized by the formation of PCNA replication foci. Cyclins undergo translocation into the nucleus and mark the transition from the G2- to M-phase. H1/H2B visualize chromosomes alignment during the M-phase. Anillin serves as a reporter visualizing cellular division. Abbreviations: CDK, cyclin-dependent kinase; PCNA, proliferating cell nuclear antigen; H1/H2B, Histone 1/2B.

These systems, however, cannot resolve all four cell cycle phases using only Cdt1 and Geminin reporters. The solution to this was addressed in the FUCCI4 system [79]. The modified version contains two additional reporters SLBP_18-126_–mTurq2 and H1–mMaroon1. The SLBP_18-126_–mTurq2 expression peaks in the S-phase and diminishes in the late S-phase, thus facilitating the identification of the S- to G2-phase transition (figure 1). Moreover, the H1 histone linker reporter is upregulated during G2-/M-phases and can be used to trace chromatin condensation and separation in the M-phase (figures 1 and 2). Even though FUCCI4 discriminates all four cell cycle phases compared with the original FUCCI and visualizes karyokinesis via histone separation, it cannot distinguish cell division from endomitosis. It would, nonetheless, be interesting to test the functionality of the FUCCI system in transgenic animal models, as it could potentially visualize differences in CM karyokinesis throughout development.

More recently, fluorescent protein lifetime (LT) properties have been exploited for the multiplexed imaging of fluorescently labelled targets within the FUCCI system. FUCCI-Red was developed to perform cell cycle analysis using a single fluorescent channel [80]. In FUCCI-Red, two red fluorescent proteins with distinct fluorescence lifetimes were fused to Cdt1 and Geminin. Fluorescence imaging was subsequently used to trace cell cycle phases based on the fluorescence lifetime of these reporters [80]. Similarly, the LT-FUCCI system uses only one channel to visualize the cell cycle by implementing lifetime-based biosensors [81]. This system uses a range of fluorophores that are activated upon binding by either Cdt1–Halotag9 or Geminin–Halotag7. As the distinct fluorophore–Halotag complexes have different fluorescent lifetimes, they offer great flexibility with regards to multiplexed imaging. Multiplexed imaging can be further facilitated by the combination of photoswitchable fluorescent protein (psFP). In work by Qian *et al*., psFPs with different off-switching rates replaced fluorescent reporters in the FUCCI4 system [82]. This modified version of the FUCCI4 system visualized transitions between all four cell cycle phases while occupying only a single colour channel and leaving the remaining channels for other targets. Single colour imaging of FUCCI4 was combined with fluorescently labelled CDK2 and CDK4/6 reporters to elucidate the relationship between these proteins at different cell cycle phases [82]. This system is compatible with other variations in FUCCI systems and represents a powerful approach to studying the interaction between multiple targets of interest throughout the cell cycle.

### 4.2. Localization-based systems

While the abovementioned cell cycle reporter systems depend on the fluorescent intensity, degradation kinetics or fluorescent lifetime of reporters, the change in cellular localization of some reporters during the cell cycle provides additional information that can be used to determine transitions more precisely.

Several members of the CDK/cyclin family, one of the main regulators of the cell cycle, compartmentalize based on the cell cycle phase and have hence been used to monitor cell cycle progression. The Cyclin B reporter was shown to visualize the transition from the G2-phase to the M-phase, as it undergoes translocation from cytoplasm to the nucleus before the onset of mitosis [67,83,84]. Much like Cyclin B, Cyclin A2 can potentially be used as a live cell cycle reporter, as its localization is stage-dependent. Its expression in the nucleus increases from the mid G1-phase, becoming highest in the late S-phase, until the onset of the mid M-phase, in which Cyclin A2 subsequently disperses from the nucleus into the cytosol [85]. By contrast to the direct visualization of fluorescently tagged cyclins, CDK-based cell cycle reporters are based on CDK activity. These kinases are activated upon binding to cyclins and are hence active during specific cell cycle phases. For example, CDK2 and CDK4/6 activities have been used to track cell cycle progression [82,86,87] (figure 2). As cyclins and CDKs are expressed at low levels in (human iPSC-derived) CMs, an increase in their expression levels would strongly suggest increased cell cycle activity. In addition, the localization of these proteins could provide valuable information with regard to the exact cell cycle phase of CMs [14,22]. Importantly though, transitions between cell cycle phases are often not regulated by only one, but rather by the summed activity of a combination of multiple cyclins and CDKs [88]. Hence, the activity of an individual reporter might not give conclusive results. Another potential disadvantage of live CDK/cyclin reporters is their functional diversity, as many cyclins and CDKs have other cellular functions besides cell cycle regulation, which might be compromised upon fluorescent labelling.

The visualization of intricate cell cycle events such as DNA replication, chromatin condensation and contractile ring formation is a more robust approach to assess cell cycle progression. For example, fluorescently labelled PCNA can be used to track cell cycle progression [84]. The distribution width of PCNA is narrow during the G1- and G2-phases due to the absence of replication foci. At the early onset of the S-phase, the distribution width increases with the formation of replication foci, reaching the peak signal at the end of the S-phase. The transition from the G2- to M-phase is characterised by a reduced PCNA signal as it spreads into the cytoplasm due to nuclear envelope breakdown (figure 2). The sequestration of the fluorescent signal within the nucleus marks the transition from the M- to G1-phase, accompanied by the reformation of the nuclear envelope [84]. As all four cell cycle phases show a distinct localization of PCNA, only one fluorescent channel is needed to assess cell cycle progression, allowing the multiplexed imaging of other fluorescently labelled proteins [84]. Other studies have used fluorescently labelled H2B histone or H1 histone linker to trace chromatin condensation, alignment and separation in the M-phase in combination with several fluorescent systems [79,83,89–91]. Similarly, the separation of DNA during the M-phase was also visualized by a Ki-67 reporter developed by Miller *et al*. In combination with the complete lack of Ki-67 in the G0-phase, this reporter might be useful to study both karyokinesis and quiescence in CMs [50] (figure 2).

Despite numerous fluorescent reporters that can indicate changes in cell cycle phases, the visualization of actual cellular division remains challenging. To resolve this caveat, the Anillin reporter was developed. Anillin marks actin–myosin contractile ring and midbody formation during cytokinesis in the late M-phase, subsequently allowing discrimination of cytokinesis from binucleation [92]. It was shown to directly visualize the division of human iPSCs and visualize CM division in murine hearts post-MI [92]. The follow-up study with the Anillin reporter showed that the symmetrical midbody localization with respect to daughter nuclei and the distance between daughter nuclei are important criteria for discriminating CM division from binucleation [58]. It would be interesting to explore the use of other fluorescently labelled cytokinetic and plasma membrane proteins as tools to discriminate between CM division and failure in cytokinesis.

While considerable progress has been made in the development and application of fluorescent reporters, including variants of FUCCI systems, the addition of localization-based reporters has provided valuable information on cell cycle phase identification. Moreover, the visualization of direct cues related to the cell cycle such as DNA condensation, separation and midbodies formation, as well as indirect cues including the symmetrical positioning of midbodies or the distance between daughter nuclei, offers novel means to distinguish cellular division from alternative cell cycle events further expanding our understanding of CM cell cycle dynamics (table 2).

### 4.3. Genetic tracing systems

A third class of fluorescent reporter systems labels proliferating CMs as the result of genetic recombination. Mosaic analysis with double markers (MADM) is based on the Cre-induced recombination of two inactive fluorescent protein genes during mitosis [93]. Consequently, cells that are labelled with either one of the fluorescent reporters can only be derived from mitosis [93]. The MADM system was further modified for clonal analysis of CMs using cardiac-specific promoter Myh6 or ɑMHC to drive Cre expression [10,94]. These systems label mononucleated CMs derived from cytokinesis with a single colour and binucleated CMs that have failed to divide with dual fluorescent colours [10,94]. In addition, AurkB reporter-based systems were recently developed to identify proliferating CMs [12,95]. These systems are based on AurkB activity to express Cre recombinase during the late M-phase and fluorescently label mitotic cells to distinguish them from non-mitotic cells. While each lineage tracing system has its strengths and limitations, collectively, they offer valuable insights into CM proliferation dynamics during heart development and regeneration. While the MADM system and AurkB-based systems have drawbacks, such as underestimating or overestimating cytokinesis, respectively, they provide valuable tools for studying CM proliferation [96]. These lineage tracing systems are not designed to monitor changes in cell cycle phases directly. Nonetheless, they can identify CMs that have divided *in vitro* as well as contribute to *in vivo* studies on CM renewal under physiological and pathological conditions (table 2).

## 5. Cell cycle visualization in advanced human cardiac organoids

Regenerating the heart through the induction of resident CM proliferation *in situ* is considered a promising therapeutic tool, as it could replace lost CMs without exogenous transplantation to the adult human heart, where endogenous regeneration is low under pathological conditions. The stimulation of CM proliferation after MI has been shown to reduce scar tissue formation and improve cardiac function post-MI [97]. Therefore, the search for pro-proliferative compounds or signalling pathways that stimulate heart regeneration has taken flight over the last decade. Interspecies differences in the CM cell cycle and the limited availability of primary human heart tissue remain, however, bottlenecks to this search. Moreover, animal models and primary tissues are incompatible with high-throughput screening often used to identify new pro-proliferative conditions. Recent advancements in the generation, expansion and cardiac-directed differentiation of human iPSCs have circumvented many of these issues and have resulted in valuable cardiac models that have been widely used to study human cardiac disease [98,99] and identify pro-proliferative compounds [31,91,100].

Antibody staining-based quantification of CM proliferation in high-throughput screening remains, however, laborious and costly, illustrating the need for alternatives. Live cell cycle reporter systems, integrated with existing human iPSC-based culture models and high-throughput screening platforms, represent a valuable alternative to evaluate CM cell cycle status. Such platforms would benefit from the functionalities of commercially available high-throughput imaging systems, which allow continuous tracking of cellular morphology, confluency and quantification of cell cycle reporter expression [101,102]. This compatibility opens avenues to conduct time-course experiments over several days to investigate the kinetics of the CM cell cycle and to identify optimal therapeutic windows. Human and mouse iPSC-derived CMs have been used to identify pro-proliferative drugs, transcription factors and miRNAs [31,100,103,104]. Moreover, recent developments in CRISPR-based gene silencing and/or activation screening strategies may provide additional insights into relevant genes responsible for CM cell cycle control and progression [105,106]. Despite the fact that two-dimensional (2D) human iPSC-derived CMs are widely used to model the heart, limitations regarding the relative immaturity of these CMs as well as the low cellular complexity have limited the predictive value of this model [107].

Over the past decade, several three-dimensional (3D) models were developed, such as cardiac organoids, engineered heart tissues and cardiac microtissues [108–112]. Compared with 2D cultures, these models promote the maturation of CMs and more closely recapitulate physiological environments such as stiffness, perfusion and the presence of other cell types [108,113]. Technical challenges associated with antibody staining in 2D cultures are, however, even more pronounced in 3D cardiac organoids: there is often incomplete penetration of the antibodies into the inner layers of the organoids and significant background staining as a result of the dense extracellular matrix [114]. Moreover, most 3D cardiac organoids, such as cardiac microtissues, are cultured non-adherently, which often results in a loss of tissues when numerous (automated) washing steps are required for fixation, clearance and antibody staining. If compatible with automated screening platforms, 3D cardiac models offer flexibility and scalability for drug discovery [115]. Together with these 3D models, live cell cycle reporters could offer a cost- and time-effective alternative to traditional antibody staining against cell cycle markers.

**Figure 3.**
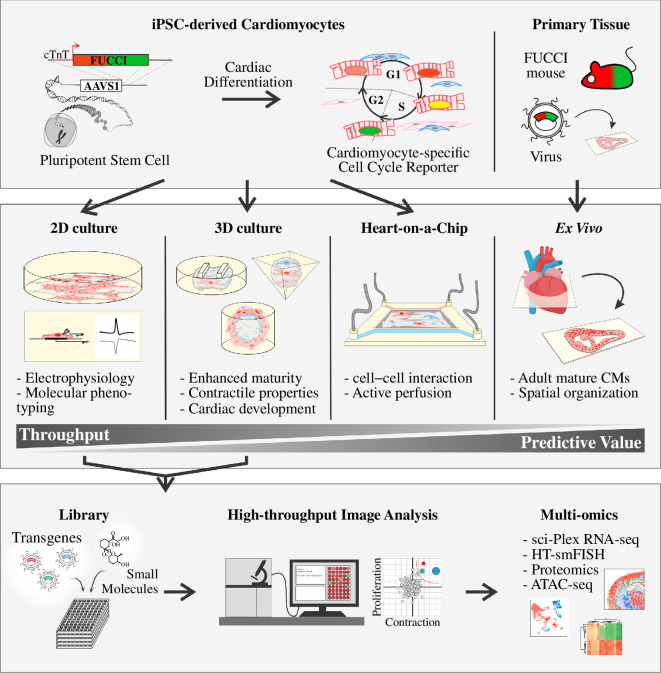
Applications of cell cycle visualization tools to study human and animal cardiomyocyte proliferation in real-time. Live cell cycle reporter systems, such as FUCCI, can be used in various 2D and 3D human cellmodels and animal studies. Human iPSC-based 2D and 3D cardiac models that incorporate live cell cycle reporters are compatible with high-throughput screening assays and multi-omic techniques. Abbreviations: AAVS1, Adeno-associated virus integration Site 1; ATAC-seq, assay for transposase-accessible chromatin using sequencing; cTnT, cardiac troponin T; FUCCI, fluorescent ubiquitination-based cell cycle indicator; HT-smFISH, high-throughput single-molecule fluorescent *in situ* hybridization; iPSC, induced pluripotent stem cell.

Live cell cycle reporter systems can be combined with a wide variety of 3D cardiac models to address a range of research questions (figure 3). Some cardiac organoids are, for example, primarily used to study cardiogenesis [116], and the use of live cell cycle reporters in such a system would help to understand cell cycle regulation in developing hearts. The integration of live cell cycle reporters in other organoid types will allow the assessment of CM cell cycle progression and contractility in parallel [113]. From a translational perspective, information on how drugs affect both CM proliferation and contractility can be used to predict the safety of future therapeutics. The exclusion of potentially detrimental compounds could therefore significantly reduce the costs and time of drug development.

The presence of other cell types, such as endothelial cells, cardiac fibroblasts, vascular smooth muscle cells or macrophages within 3D cardiac models will also facilitate studies focused on the effect of paracrine crosstalk on the cell cycle of CMs (figure 3). The cellular heterogeneity within these 3D models makes it, however, more challenging to assess cell cycle progression in CMs specifically. To discriminate CM cell cycle activity from that of other cell types, the generation of a cell type-specific reporter system using genetic editing methods can be useful, as was demonstrated by Murganti *et al*., who showed that the FUCCI system was integrated into one of two CM-specific *Tnnt2* alleles [31]. Alternatively, CM-specific promoter-driven cell cycle reporter transgenes can be integrated into safe harbour loci [117]. The latter might, however, require some optimization as silencing of these genomic safe harbours loci during the differentiation of human iPSCs has been reported [118].

The addition of non-myocyte cells in several 3D models helps to recapitulate the cellular crosstalk and diversity seen in native cardiac tissues. However, the spatial organization and mechanical characteristics of native tissue are not accounted for in most models. Heart-on-a-chip platforms can partially mimic the 3D extracellular environment of native myocardium and can be further modified by using tissue bioprinting, electrical and/or mechanical stimuli that promote maturation of CMs, and the inclusion of microvasculature formed by endothelial cells [109,119,120]. Genetically modified CMs carrying a live cell cycle reporter system can provide valuable insights into how external physical cues influence the cell cycle in heart-on-a-chip platforms. Additionally, they could be used in multi-organ systems to gain a better understanding of endocrine effects on CM proliferation [121,122].

Although these 3D models have considerable advantages, they still do not fully recapitulate all aspects of the native tissue microenvironment in terms of cellular complexity and spatial organization, although this may not be necessary if a model is ‘fit-for-purpose’. This limitation has nevertheless been addressed by developing methods for the isolation and culturing of slices of animal- and human-derived cardiac tissues. Cardiac slices have been isolated from FUCCI transgenic mice and have contributed to our knowledge on the cell cycle changes that occur throughout heart development [28]. However, results obtained from these murine cardiac slices are difficult to translate to the human context, due to differences in CM cell cycle behaviour, nucleation and ploidy status, stressing the need for a human-specific alternative.

## 6. Future perspectives

Live cell cycle reporter systems can serve as invaluable tools for unravelling CM proliferation dynamics, offering insights into cell cycle regulatory mechanisms. These systems overcome limitations associated with traditional antibody staining methods and exhibit compatibility across different 2D, 3D, and *in vivo* cardiac models. Moreover, recent advancements in live cell cycle reporter systems have addressed many challenges related to distinguishing cell cycle phases and have offered multiplexing capabilities. Yet, it is crucial to carefully consider the advantages and technical limitations of each system to ensure appropriate applications that fit a specific research question.

Despite the development of advanced CM-specific live cell cycle reporters, recent technological advances offer new opportunities to further enhance their utility. Future research could, for example, benefit from a diversification of cell type-specific cell cycle reporters. Recent single-cell RNA-sequencing data have led to the identification of new CM subpopulations with distinct transcriptional profiles [123]. The integration of multiple single-colour reporter systems (e.g. FUCCI-Red and LT-FUCCI) under the control of (newly identified) cell type-specific promoters would allow the simultaneous monitoring of cell cycle progression in several distinct cell types or subpopulations. This could be used to understand how the cell cycle of CMs is dependent on that of other surrounding cells or the differences in regenerative potential between subpopulations. Moreover, this approach could benefit the search for compounds that selectively activate proliferation in CMs without affecting other cell types.

Additional improvements might shift the focus from the CM cell cycle towards a more inclusive measure of functional cardiac regeneration. Although the induction of CM proliferation is often used as a proxy for increased regenerative potential, the contractile properties of these newly formed CMs might actually be equally important from a therapeutic perspective. Cellular division is, for example, often accompanied by CM de-differentiation, sarcomere disassembly and reorganization, compromising its contractile properties [124, 125]. The inclusion of a sarcomere reporter, e.g. ɑ-actinin, troponin I or titin [126–128], could potentially be used to assess changes in sarcomere organization and contractility during the cell cycle and after cellular division in detail, thereby providing important insights into potential deleterious effects of pro-proliferative drugs. Designated software, such as MuscleMotion and SarcTrack [129,130], and advancements in cloud-based computational power provide the IT infrastructure for handling such imaging-based high-throughput screens to assess CM proliferation and contractility.

Altogether, we envision that the development of more advanced live cell cycle reporters, and their integration into novel cardiac model systems, will provide exciting opportunities for future studies aimed at improving cardiac regeneration. In combination with the increasing diversity of large screening libraries, like FDA-approved drugs or CRISPR guide RNAs, and the growing set of high-throughput tools to molecularly phenotype large numbers of cells or tissues in a single experiment [131–133], such systems hold great promise for advancing the high-throughput discovery of regenerative agents.

Ideally, promising cardiac regenerative agents would be best tested on primary human heart tissue that allows easy quantification of CM cell cycle progression. To the best of our knowledge, the application of live cell cycle reporters in *ex vivo* cultured human myocardial slices has not yet been reported. Several studies have, however, shown robust transgene expression in CMs within human cardiac slices transduced by viral vectors [134–136], indicating that CM-specific expression of cell cycle reporters in human cardiac slices, either through the use of cardiac-promoter-driven transgenes or cardiac tropic adeno-associated viruses, is technically feasible (figure 3). In this context, improvements in imaging methods could be important for detecting fluorescent signals from deeper tissue layers or providing high-resolution images with minimum phototoxicity over an extended period.

Human cardiac slices that express live CM cell cycle reporters would not only provide extremely valuable information about the cell cycle dynamics of human CMs within their native tissue microenvironment but may also reveal differences in the cell cycle state between healthy and diseased heart tissues. Although less suited for high-throughput screening, such an advanced system would reflect the human tissue *in vivo* situation more closely, offer high predictive value, and likely reduce the number of experimental animals used.

## Data Availability

This article has no additional data.
